# Human Stem Cells for Cardiac Disease Modeling and Preclinical and Clinical Applications—Are We on the Road to Success?

**DOI:** 10.3390/cells12131727

**Published:** 2023-06-27

**Authors:** Cátia D. Correia, Anita Ferreira, Mónica T. Fernandes, Bárbara M. Silva, Filipa Esteves, Helena S. Leitão, José Bragança, Sofia M. Calado

**Affiliations:** 1 Algarve Biomedical Center Research Institute (ABC-RI), Universidade do Algarve—Campus de Gambelas, 8005-139 Faro, Portugal; a57384@ualg.pt (C.D.C.); a57399@ualg.pt (A.F.); mafernandes@ualg.pt (M.T.F.); barbarasmsilva@gmail.com (B.M.S.); filipa.esteves@abcmedicalg.pt (F.E.); hcleitao@ualg.pt (H.S.L.); jebraganca@ualg.pt (J.B.); 2 Algarve Biomedical Center (ABC), Universidade do Algarve—Campus de Gambelas, 8005-139 Faro, Portugal; 3 School of Health, Universidade do Algarve—Campus de Gambelas, 8005-139 Faro, Portugal; 4 Doctoral Program in Biomedical Sciences, Faculty of Medicine and Biomedical Sciences, Universidade do Algarve—Campus de Gambelas, 8005-139 Faro, Portugal; 5 Faculty of Medicine and Biomedical Sciences, Universidade do Algarve—Campus de Gambelas, 8005-139 Faro, Portugal; 6 Champalimaud Research Program, Champalimaud Centre for the Unknown, 1400-038 Lisbon, Portugal

**Keywords:** human stem cells, cardiovascular diseases, iPSC, disease modeling, cell therapy

## Abstract

Cardiovascular diseases (CVDs) are pointed out by the World Health Organization (WHO) as the leading cause of death, contributing to a significant and growing global health and economic burden. Despite advancements in clinical approaches, there is a critical need for innovative cardiovascular treatments to improve patient outcomes. Therapies based on adult stem cells (ASCs) and embryonic stem cells (ESCs) have emerged as promising strategies to regenerate damaged cardiac tissue and restore cardiac function. Moreover, the generation of human induced pluripotent stem cells (iPSCs) from somatic cells has opened new avenues for disease modeling, drug discovery, and regenerative medicine applications, with fewer ethical concerns than those associated with ESCs. Herein, we provide a state-of-the-art review on the application of human pluripotent stem cells in CVD research and clinics. We describe the types and sources of stem cells that have been tested in preclinical and clinical trials for the treatment of CVDs as well as the applications of pluripotent stem-cell-derived in vitro systems to mimic disease phenotypes. How human stem-cell-based in vitro systems can overcome the limitations of current toxicological studies is also discussed. Finally, the current state of clinical trials involving stem-cell-based approaches to treat CVDs are presented, and the strengths and weaknesses are critically discussed to assess whether researchers and clinicians are getting closer to success.

## 1. Introduction

Cardiovascular diseases (CVDs) are the leading cause of death worldwide, contributing to approximately 32% of all deaths [1]. CVDs are a broad spectrum of diseases commonly affecting the heart and blood vessels; they have a complex etiology and are influenced by nonmodifiable and modifiable risk factors [2,3]. According to the World Eurostat, EUR 84 million was allocated for hospitalization of patients suffering fromcardiovascular issues across EU members in 2019 [4,5]. Thus, heart diseases and related healthcare represent a massive social and economic burden. From a pathophysiological point of view, the heart is very vulnerable to hypoxia and has a very limited ability to regenerate. Upon a lesion, lost cardiomyocytes are not fully replaced by new functional cells, and instead, necrosis and unfavorable heart tissue remodeling (fibrotic scar tissue) occurs and leads to further functional loss [6,7] Currently, the treatment of CVDs relies mainly on traditional pharmacotherapy and surgery. However, although effective in relieving the symptoms and reducing mortality, long-term pharmacotherapy induces renal failure, rhabdomyolysis, hemorrhages, and hepatotoxicity [1]. On the other hand, cardiac surgeries are complex procedures and often entail postoperative complications [8]. Therefore, novel strategies to reliably diagnose, refine the treatment, and, if possible, prevent CVDs are urgently needed and a great challenge for patients, health professionals, and researchers.

The discovery of human stem cells with therapeutic potential opened a door not only to improve cardiac regeneration but also has provided a model to study the molecular mechanisms associated with CVDs and the identification of new drugs [9,10]. Biomedical research and the potential use of human stem cells that can be manipulated to originate new cardiac functional cells to repair a damaged heart have attracted much attention in the past two decades. Anumber of clinical trials involving stem cell therapies to treat CVDs has surged, and a significant number of additional trials are still underway [11]. However, even though myocardial infarction (MI), and chronic heart failure are the most common CVDs, conditions such as heart failure, left ventricle dysfunction, and ischemic heart disease (IHD) are the ones that seem to be their primary targets in clinical trials [12]. Stem cell therapy represents one of the most important and necessary steps in cardiovascular medicine, and their related clinical trials have produced some encouraging results that suggest these therapies are well tolerated. Unfortunately, stem-cell-based therapeutic strategies to efficiently improve heart repair have not been fully established yet. The determination of the ideal cell type, the cell dose, the timing for cell delivery, as well as the route of administration are factors that can influence stem-cell-based therapies. In this review, we address the current state of the art on the use of human stem cells in disease modeling, drug toxicity, and regenerative medicine for CVDs. We also discuss the main successes and limitations of stem cell transplantation for CVD therapy.

## 2. Human Stem Cell Types and Sources for CVD Research and Treatment

Stem cells are a type of undifferentiated or partially differentiated cells that display two special features: (i) self-renewal capacity (during cell division, stem cells originate daughter cells identical to their parental cell) and (ii) the ability to generate differentiated cells [13]. The differentiation potential of a stem cell into different cell types is highly dependent on its potency. Thus, stem cells can be categorized as totipotent (the zygote, which can generate a full organism, including embryonic and extra-embryonic tissues), pluripotent (which can generate the three embryonic germ layers), multipotent (which can generate several cell types but are committed to a specific cell lineage), oligopotent (which originates fewer cell types than multipotent stem cells), and unipotent (which can only originate one specific cell type). Regarding their origin, stem cells can be found in the embryo (embryonic stem cells—ESCs) or in adult tissues (adult stem cells—ASCs) [10].

**Figure 1:**
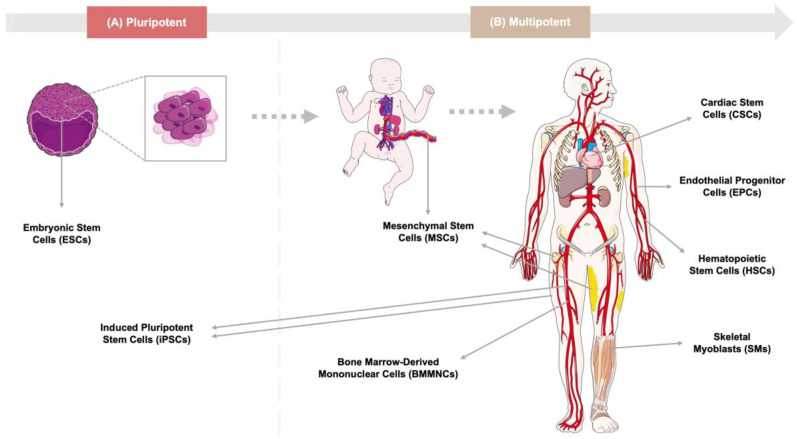
Sources and types of human stem cells. (**A**) Pluripotent cells, such as embryonic stem cells (ESCs) and induced pluripotent stem cells (iPSCs) are obtained from the inner cell mass of the blastocyst and by reprogramming of somatic cells (e.g., collected from dermal skin fibroblasts or peripheral blood), respectively. They can generate cells and tissues derived from the three embryonic germ layers. (**B**) Several types of multipotent stem cells are found in adult tissues. These have a more restrictive differentiation potential than pluripotent stem cells. Examples of multipotent stem cells currently being used in CVD therapy include (i) mesenchymal stem cells (MSCs), which can be derived from different tissue sources such as bone marrow, fat adipose tissue, and umbilical cord; (ii) bone-marrow-derived mononuclear cells (BMMNCs); (iii) cardiac stem cells (CSCs); (iv) endothelial progenitor cells (EPCs); (v) hematopoietic stem cells (HSCs); and (vi) skeletal myoblasts (SMs). This figure was partially drawn by using pictures from Servier Medical Art. Servier Medical Art by Servier is licensed under a Creative Commons Attribution 3.0 Unported License (https://creativecommons.org/licenses/by/3.0/, accessed on 28 May 2023).

Several studies have shown that human stem cells exert beneficial effects on cardiac regeneration. Here, we focus on cells that have shown the most promising potential, such as skeletal myoblasts (SMs), bone-marrow-derived mononuclear cells (BMMNCs), hematopoietic stem cells (HSCs), endothelial progenitor cells (EPCs), mesenchymal stem cells (MSCs), cardiac stem cells (CSCs), embryonic stem cells (ESCs), and induced pluripotent stem cells (iPSCs). The types and sources of stem cells currently being used for the treatment of CVDs are summarized in Figure 1.

### 2.1. Skeletal Myoblasts (SMs)

SMs are a group of satellite progenitor cells that participate in skeletal muscle injury repair. Apart from their embryonic and morphologic similarities with heart muscle cells, SMs can be easily obtained from autologous muscle biopsies and present rapid expansion in vitro, ischemic tolerability, low risk of tumorigenicity, and, more importantly, myogenic differentiation capacity [14,15]. For these reasons, SMs were the first cells clinically tested in myocardial repair [16]. However, despite reported improvements in cardiac performance in patients transplanted with SMs, many of the patients experienced ventricular arrhythmias due to the lack of electromechanical integration of SMs with resident cardiomyocytes cells [15]. For this reason, SMs have lost popularity for cardiac applications.

### 2.2. Bone Marrow-Derived Mononuclear Cells (BMMNCs)

Since the late 1990s, bonemarrow-derived cells have demonstrated their ability to migrate to injured tissues and promote their regeneration [16,17,18]. Bone-marrow-derived stem cells can be found in the peripheral blood [19] and are generally divided into two major populations: HSCs and non-HSCs, the latter including MSCs. More details about HSCs and MSCs are addressed later in this review. Their abundance and accessibility allow autologous implantation without previous expansion, which prevents stem cell differentiation and immune rejection and promotes cellular migration to the affected tissues [20]. A recent meta-analysis showed improved myocardial performance after BMMNCs transplantation and reduced rehospitalization and reinfarction rates, although no effect on reduction of cardiovascular-related death was observed [21].

### 2.3. Hematopoietic Stem Cells (HSCs)

HSCs constitute a subpopulation of BMMNCs residing in a specialized bone marrow environment known as the HSC niche [21], which are multipotent. They originate all blood cells and a subpopulation of pro-vasculogenic EPCs and are characterized by the presence of CD133 and/or CD34 surface markers [22]. Although HSCs can be autologously transplanted by straightforward and standardized isolation protocols, and their safety was clinically tested, their limited abundance and differentiation potential compromised their therapeutic use [1,22].

### 2.4. Endothelial Progenitor Cells (EPCs)

EPCs are a mixed population of cells that can be originated from HSCs of the bone marrow (H-EPCs) or nonhematopoietic tissues (non-H-EPCs) [23,24]. H-EPCs are a subpopulation of HSCs with pro-vasculogenic properties that enter circulation under specific stimuli. Non-H-EPCs are found in the peripheral blood or other tissues and can acquire an EC-like phenotype after successive cultures [24]. EPCs can be distinguished from HSCs by the presence of CD31 and VEGFR2 endothelial surface markers [23,25]. It is known that several CVDs are closely related to endothelial dysfunction [23]. Due to their homing capacity, which allows them to reach the injured tissue after an insult, and the paracrine signaling mediated by these cells, which stimulate the proliferation of the endothelium surrounding the damaged tissue, EPCs have been considered as a cell therapy strategy for CVDs [23,24]. Although some studies have shown the effectiveness of EPCs in improving heart function [26,27], their effect is limited by their reduced differentiation capacity [28].

### 2.5. Mesenchymal Stem Cells (MSCs)

MSCs are a heterogeneous population of adherent, fibroblast-like multipotent cells, which can differentiate into several cell types of the mesodermal lineage, such as osteoblasts, adipocytes, and chondrocytes [29], and should express CD29, CD44, CD73, CD105, CD106, and CD166 surface markers but not the hematopoietic and endothelial surface markers CD11b, CD14, CD31, CD34, CD45, or HLA-DR [24,29]. MSCs were firstly isolated from the bone marrow, but they can also be found in the placenta, amniotic fluid, cord blood, menstrual blood, and adipose tissue, which are more attractive because these involve a less invasive isolation procedure as compared to isolation from the bone marrow [1,29]. In addition, MSCs are more abundant in the adipose tissue than in the bone marrow [30]. Due to their immunomodulatory and anti-inflammatory properties, MSCs have been the most-studied stem cells for the treatment of cardiac injury [29]. Apart from their direct effect on cardiac tissue repair and regeneration [31], MSCs may also play important roles through the secretion of trophic factors [29], which improve cardiac function by tissue injury reduction, inhibition of fibrotic remodeling, angiogenesis, activation of host tissue stem cells niches, and reducing inflammation. Moreover, several studies show that MSCs can escape immune surveillance [32], which allows the transplantation of both autologous and/or allogenic cells [33]. Despite the potential of MSCs, clinical trials have shown conflicting results on their effect in the treatment of CVDs [34]. These differences can be due to several causes, including the protocols used for the manipulation of the MSCs, which might influence the viability and therapeutic potential of the cells, the administration route used to transplant them, as well as the intrinsic differences in functional cardiac parameters and severity among participants [34].

### 2.6. Cardiac Stem Cells (CSCs)

For decades, it was universally accepted that cardiomyocytes (CMs) are post-mitotic cells, thus exhibiting a very limited regenerative ability after birth. This concept has been changed recently. Bergmann and coworkers showed that CMs did renew, however, with a very low turnover rate. Indeed, they showed that approximately 50% of the CMs are exchanged during a lifespan, thus supporting the existence of lifetime repopulation of CMs [35].

The origin of CSCs is still unknown. However, researchers have been able to isolate them from heart tissue and expand them in vitro for cardiac therapy in vivo. Different populations of CSCs have been identified and investigated in preclinical studies, such as cardiac Sca-1^+^ cells, cardiac c-kit^+^ cells, cardiac side-population cells, epicardium-derived cells, cardiosphere-derived cardiac cells, and cardiac islet-1^+^ cells [24]. Although some studies have shown that the administration of CSCs preserved heart function and reduced adverse structural remodeling [36], stimulated angiogenesis and improved left ventricular function [37], and induced a dose-dependent functional benefit in mouse models of MI [38], their reduced number (1 CSC per 10,000 CMs) limits their use [24].

### 2.7. Embryonic Stem Cells (ESCs)

ESCs are pluripotent stem cells obtained from the inner cell mass of the blastocyst and are capable of generating the three germ layers composing an embryo: endoderm, mesoderm, and ectoderm. They were firstly isolated and cultured from human blastocysts by Thomson and coworkers in 1998 [39]. ESCs display several advantages over adult stem cells (ASCs), including the pluripotent differentiation potential, which is virtually unlimited in vitro due to their self-renewal capacity, and they can generate structural and functional-like CMs [22]. Although ESC-derived CMs were shown to improve cardiac function in rats [40], pigs [41], and even non-human primates [42], the ethical concerns, the poor engraftment and survival of the transplanted cells, and the risk of immune rejection and teratoma formation due to incomplete differentiation discourage the use of ESCs in clinical trials [9].

### 2.8. Induced Pluripotent Stem Cells (iPSCs)

iPSCs are a special class of pluripotent stem cells that originate from somatic cells through the exogeneous co-expression of OCT4, SOX2, KLF4, and c-MYC transcription factors in a process called reprogramming [43]. These cells were originally generated from fibroblasts, but currently, several researchers have efficiently reprogrammed other somatic cells such as keratinocytes [44,45], peripheral blood mononuclear cells [46,47], or squamous epithelial cells collected from urine [48,49].

The iPSCs have several advantages over ESCs since they can be generated from several tissue sources and can be administered autologously, with no major ethical concerns associated. In addition, similarly to ESCs, iPSCs can differentiate into cells from the three germ layers including CMs, and due to their self-renewal capacity, they represent an unlimited source of CMs [50,51]. However, although in vivo studies using iPSCs-derived CMs (iPSC-CMs) having shown improved cardiac function and reduced infarction size in animal models of MI [52], functional analyses revealed that these cells are immature and more similar to embryonic CMs [53]. Apart from this, the risk of tumorigenicity and immune rejection due to genomic instability and lack of standardized and inefficient differentiation procedures, which culminate in low numbers of pure and mature CMs, limit their translation into clinical practice [1].

## 3. Modeling Human Cardiovascular Diseases Using Stem Cells

The human body is a complex system comprised of hundreds of cell types interacting with each other. Overall, ~30% of the adult heart is comprised of contractile CMs, and the remaining is composed of cardiac endothelial cells (ECs), vascular stromal cells, and cardiac fibroblasts (CFs) [54], where one or more of these cells can be affected in a variety of ways possibly leading to CVDs. In patients diagnosed with the same disease, the underlying biological and molecular pathways may differ even in those sharing identical symptoms. Therefore, disease models that can recapitulate the endogenous mechanisms of the human organism in vitro are crucial in providing important insights into the molecular basis of cardiovascular development, disease progression, and pathogenesis, which allow important therapeutic advancements [55,56]. Studies in animal models, more specifically in genetically modified mice for knockout or disease-specific mutations, have been essential for clarifying the general principles of heart development and disease [57,58]. Mice have been widely used as a model of CVDs due to their relatively high homology with the human genome, well-established sophisticated methods of genetic manipulation, and ease of breeding and maintenance in the laboratory [59,60]. These models, however, have limited suitability and may not provide comprehensive knowledge to address human-specific aspects of development, disease, and therapy [58]. Species-specific differences in physiology, metabolism, and genetic expression patterns represent obstacles in disease modeling that can lead to inefficient and inaccurate results when translated from murine preclinical studies to human clinical trials [60,61]. For these reasons, human-based models are particularly important for cardiovascular research [62]. However, there is still a notable lack of human cardiac in vitro models that can sufficiently recapitulate cardiogenesis [63,64,65].

### 3.1. Advantages and Disadvantages of Stem Cells to Model CVDs

To model complex diseases, it is necessary to establish robust differentiation protocols that allow the isolation of a large number of cell populations that can be fully differentiated into the functional cells and tissue types of a human adult heart [66].

#### 3.1.1. Mimicking Heart Development to Obtain Cardiac Cells

Heart development is a dynamic, three-dimensional process controlled by an intricate network of signals and gene transcription [67]. The ability of stem cells to be differentiated into specific human cell types of interest allow the selection of compounds that preferentially modulate the activity of those cells with the limited activity of other cell types [68]. Protocols for in vitro differentiation or maintenance of CSCs from either ESCs or iPSCs rely primarily on simulating the heart’s naturally occurring signaling microenvironment by using growth factors, epigenetic modifiers, neurotransmitters, and extracellular matrix proteins, and these need either the activation or inhibition of specific signaling pathways [1,69]. In particular, pluripotent stem cell differentiation into CMs is achieved by the activation of WNT, ACTIVIN/NODAL, and BMP signaling to induce mesoderm specification (through an epithelial-to-mesenchymal transition phenomenon), followed by WNT signaling inhibition to generate cardiac mesoderm (mesodermal specification) and eventually cardiogenic differentiation (cardiac-specific progenitors), followed by selection for cardiac markers (structural genes encoding for sarcomeric-related proteins of terminal differentiated CMs) [70,71]. This bi-phasic modulation of WNT signaling (i.e., activation followed by inhibition) is enough to induce cardiac specification [69]. In addition, the timing in which those key factors and auxiliary molecules are added seems to be critical in stem cell differentiation [1].

The development of differentiation protocols of ESCs towards various cardiovascular cell types such as CMs, ECs, and smooth muscle cells (SMCs) has been endorsed due to their differentiation potential [64]. ESC-CMs express several structural proteins, actin and actin-regulatory proteins, vascular collagens, as well as gap junctions. These CMs also display stable and spontaneous pacemaker activity, functional syncytium, and conduction properties. In addition, electrophysiological recordings match all the three action potential shapes found in the adult heart, namely nodal-, atrial-, and ventricular-like, and most ion currents that compose them [64,72]. ESC-ECs offer potential therapeutic implications, including the repair of ischemic tissues and tissue engineering of vascular grafts, as they can spontaneously organize vessel-like structures in vitro in a pattern that recapitulates embryonic vascularization [66]. However, as discussed before, the ethical concerns and the safety issues associated with the use of ESCs limits their application in patients with CVD [73].

#### 3.1.2. CVD Modeling Using iPSCs

The generation of iPSC-CMs is of growing interest, as they have the capacity to recapitulate the mechanisms of in vivo cardiac development in an in vitro setting [74,75], demonstrating the ability to form mixed populations of cell subtypes and exhibiting most of the individual, cardiac-specific ionic currents/electrophysiological properties [62,76]. Consequently, the identification of selection markers is crucial for the purification of CM progenitors [14].

Based on these and other characteristics previously addressed, iPSC-derived cells can recapitulate cellular phenotypes of monogenic disorders and polygenic/complex diseases when combined with gene-editing technologies [74,77]. This combination allows the study of mutations and SNPs (single-nucleotide polymorphisms) under the same genetic background, contributing to the analysis of patient-specific disease phenotypes, which represents a promising tool for investigating the correlation of differences in gene expression and genetic variations among individuals [77]. On the other hand, using iPSC-based models for complex diseases remains challenging, considering that many CVDs are often characterized by late onset, involving multiple gene variants, and in some cases also different tissues and cell types. For this reason, it is important to identify the appropriate control lines, for example, by comparing the patient’s iPSC-derived CMs (iPSC-CMs) to those from a healthy sibling, to limit genetic variability [57,78].

iPSC-CMs have been used to model CVDs such as left ventricular non-compaction (LVNC), which is caused by a structural abnormality of the left ventricle where the myocardium appears hypertrabeculated [50,79], as well as hypoplastic left heart syndrome, characterized by severe underdevelopment of the left ventricle (LV), mitral valve, aortic valve [79,80], as well as ascending aorta and atrial or ventricular septal defects [79]. However, using models of iPSC-derived cardiovascular cell types does not always recapitulate human disease phenotypes. Considering that congenital heart disease is often originated from abnormalities in cardiovascular cell differentiation, specification, and migration, in vivo, these models tend to replicate these mechanisms that originate developmental malformations in vitro [79]. Another major downside of iPSC-derived CMs is, as stated above, their heterogeneity and immaturity, considering that they are structurally, functionally, and genetically similar to embryonic CMs rather than adult CMs [62,81,82]. Indeed, iPSC-derived CMs display an immature sarcomeric architecture, high proliferation rates, spontaneous contraction due to high pacemaker currents, immature sarcoplasmic reticulum, and round/oval rather than rod-like shapes [53]. Despite this, it has been shown that between day 52 and 56 of iPSC-CM differentiation, the electrophysiological properties can stabilize withing the ranges of adult human tissue [83]. This emphasizes how the current differentiation protocols are not very efficient and points to important steps that still need to be solved since a highly pure and mature CM population is needed for cell therapy and tissue-engineering purposes [82]. Several approaches have been attempted for pluripotent stem cells-CMs maturation, and some have succeeded. Some of these include the addition of thyroid hormone, a thick layer of Matrigel^®^, long-term cultivation, 3D scaffold-based cultures, or in vitro engineered heart tissues (EHTs), in which the differentiation is carried on microfabricated polydimethylsiloxane 3D pillar array patches to enhance the expression of genes involved in cardiac contractile function and to promote CM elongation, orientation, alignment, and conduction velocities [84,85,86].

### 3.2. Two-Dimensional (2D) vs. Three-Dimensional (3D) In Vitro Models

**Figure 2:**
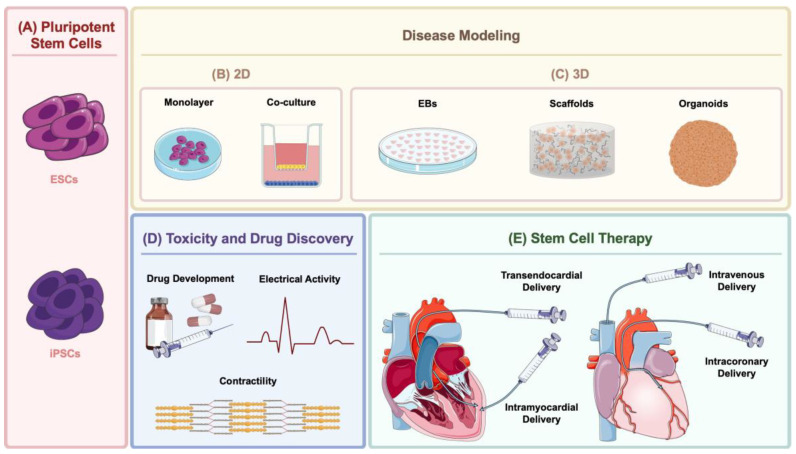
Stem cells in disease modeling, toxicity studies, drug discovery, and cell therapy for CVDs: Pluripotent stem cells (**A**), including ESCs and iPSCs, are often used for the modeling of CVDs. They can be differentiated in cardiac cells both in 2D (**B**) or 3D cultures (**C**), which can be supported by the inclusion of different scaffolds and matrices. The differentiated cells can be used for drug discovery and toxicity studies (**D**) and for cell therapy purposes through transendocardial, intramyocardial, intravenous, or intracoronary delivery (**E**). This figure was partially drawn by using pictures from Servier Medical Art. Servier Medical Art by Servier is licensed under a Creative Commons Attribution 3.0 Unported License (https://creativecommons.org/licenses/by/3.0/, accessed on 6 April 2023).

To better understand the onset, course, and molecular mechanisms of various diseases and to develop new therapeutic strategies, it is crucial to develop new, human-relevant disease models. The protocols used for pluripotent stem cell differentiation into CMs can be divided into two different groups based on 2D or 3D methods. In 2D differentiation protocols, two main approaches have been developed: (i) monolayer cell culture or (ii) co-culture of two or more cell types (Figure 2B). In 3D differentiation protocols, embryoid bodies (EBs) formation, microtissues with scaffolds, and, more recently, human heart organoids (hHO) have been established (Figure 2C).

#### 3.2.1. Two-Dimensional Models for CVD

The monolayer technique involves the use of pluripotent stem cells (ESCs or iPSCs) (Figure 2A) cultured in a feeder-free system that is induced to differentiate into a specific cell type, such as CMs (Figure 2B). For that, a defined serum-free medium with growth factors and specific small molecules is used to induce the cells to turn the right commitment [73,87]. This method is user-friendly, more controlled, reproducible, cost-effective, and allows higher homogeneity in cell growth and development as well as better access to nutrients and growth factors [88]. Another positive point about monolayer culture is the avoidance of extra purification steps to generate functional CMs [1,74,87], as it is highly efficient and shows a high-percentage yield of purified CMs (up to 98% functional cTNT^+^ CMs) [1,74,87,89]. It has been commonly used to study cellular mechanisms underlying specific cardiac processes [88], such as calcium handling and contractility [62], and for drug testing, discovery, and development [60]. This technique has also been used to model cardiovascular conditions such as reduced contractility and impaired desmosome due to loss-of-function mutations in *PKP2*, which causes arrhythmogenic cardiomyopathy (AC), using isogenic iPSC-CMs. In this study, the isogenic set of iPSC-CMs did recapitulate AC pathology and provided a rapid and convenient cellular platform for therapeutic development [90]. However, because a disease is often not due to just a single cell type but multiple, it is necessary to use more than one type of cells in culture to recapitulate more efficiently what happens in vivo. This is possible using co-cultures, where different cell types share the same environment (Figure 2B).

By using co-culture set-ups, it is possible to study the crosstalk between various cells and between the cells and the extracellular matrix (ECM). In these systems, two main cellular groups, i.e., the target cells, which are responsible for mimicking the tissue under study, and the assisting cells, which are responsible for creating an environment by the secretion of signaling molecules or production of ECM and assisting the other group of cells to adhere, proliferate, and differentiate, are cultured simultaneously with some degree of contact. Co-cultures can be indirect or direct. On an indirect co-culture, there is a physical separation of the cell types in use, but they share a common medium; thus, the interaction is based on soluble factors within the same culture environment. This allows the study of cell–cell crosstalk through paracrine signaling, which can be performed also with conditioned media, where there is a transfer of the assisting cells’ supernatant to the target cells. Another indirect co-culture method is performed by using transwells, in which the different cell types are both present but physically separated by a membrane filter, preventing direct cell-to-cell contact. Then, one cell type is cultured on the insert filter and the other one below it on the bottom of the culture plate. The variability of membrane filter types and the pore sizes facilitates a range of applications such as drug transport, permeability studies, or cell–cell/cell–substrate interactions within a co-culture system [91]. In direct co-cultures, different cell types are mixed in the same culture, allowing physical contact, thus contributing to a better cell–cell and cell–ECM level of communication [91,92]. Protocols using co-cultures require few cells, are simple and rapid and allow to obtain CMs in sufficient quantity and quality [74], and can be used to induce structural and functional maturation of iPSC-CMs [93]. Some studies have used iPSC co-culture with somatic cells to induce the differentiation since the morphogens secreted from assisting cells can improve the differentiation of target cells into the desired lineage [94]. iPSC-derived cell types can be grown together with other cells mimicking the tissue of interest. Endothelial cells are the most widely used cell type for co-culture models, considering their role in coating the blood vessels, supplying nutrients and oxygen to the tissues, and secreting pro-cardiac cytokines. Other studies also use fibroblasts co-cultured with iPSC-CMs, which are known to alter CM hypertrophy, function, and gene expression [61,89,95,96,97]. The first in vitro human heterotypic model of myocardial ischemia/reperfusion (IR) injury was created using human CPCs and iPSC-CMs in co-culture in transwells [98]. This study demonstrated the importance of communication between these two cell populations and the role of cytokines, as iPSCs-CMs death due to IR injury is prevented in co-culture conditions due to the paracrine effects of CPCs [98].

#### 3.2.2. Three-Dimensional Models for CVD

The human body has a very complex three-dimensional (3D) structure and physiology, and although using cell culture dishes to analyze cell subtypes has been a resource greatly used over time to study lineage-specific disease mechanisms, cells in the human body do not function in mono-lineage isolation, and diseases display a multi-lineage or multi-systemic impact, with just a single affected cell type triggering a domino effect that influences downstream pathophysiology in diverse tissues [61]. Thus, introducing in vivo complexity into a tissue culture system is required to address the challenge of modeling a tissue microenvironment [66]. Because of this complexity, a new generation of models is being developed to meet the escalating demand for multi-lineage analysis. These models consist of 3D culture systems (Figure 2C), where the more commonly used are based on EBs formation. With 3D systems, tissue microenvironmental cues are retained, thus providing a more natural tissue environment to study cell function, which is not possible to reproduce with 2D cell culture systems [99].

EBs are 3D cellular aggregates usually obtained when stem cells are cultured in suspension only, with medium and fetal bovine serum as a source of inducing factors (Figure 2C) [86,100]. The cells that compose the EBs are able to differentiate in all cell types deriving from the three primary germ layers [101]. This allows to recapitulate the growth factor gradients and the cell–cell interactions that normally occur in the human embryo, resembling early steps of development. Later on, these structures, similar to the morula stage, show contraction and cardiac markers [1,14,64,102]. However, as they grow, it becomes more difficult for nutrients and oxygen to diffuse into the core of these structures [103]. EBs can be also very heterogeneous in reproducibility, structure, and composition, so critical parameters have been optimized in protocols for the homogenization of EB factors such as shape and size. Some factors such as the medium and serum, the starting number of cells that compose the aggregates, the cell line used, as well as the timepoints during the protocols are extremely important to homogenize the size of the EBs and to improve differentiation [1].

EB formation can be achieved in non-adherent conditions by methods such as suspension culture, the hanging drop method, bioreactor culture, spinner flask, and microwell technologies, followed by plating EBs on tissue culture plates coated with gelatin and supplemented with specific growth factors or differentiation-related cofactors. Regarding the first one, spontaneous aggregation of EBs happens, and these have heterogeneous sizes and shapes. This method has some advantages, as it is easy, fast, effective, scalable, and user-friendly. The hanging drop technique is controlled, and the EBs are uniform in size, but it presents some technical difficulties, especially related to pipetting or medium changing. Using bioreactor cultures as a homogenous supply environment to sustain EB generation and the induction of these to differentiation is common and used to improve direct differentiation. Bioreactors have their benefits, such as simple scale-up EB formation, manageable culture parameters, and well-functioning treatments. Unfortunately, their large volumes lead to expensive and difficult procedures [1].

Based on their general characteristics, EBs can be used for cardiotoxicity evaluations, regenerative medicine studies, tissue engineering, and transplantation therapy [88,103]. More specifically within the cardiac branch, they have been used to describe phenotypic properties of CMs derived from human ESCs by manipulating them to form EBs containing regions of spontaneous contraction, with the purpose of studying human cardiac tissue development [100]. This study demonstrated that the human cardiomyocytes produced from ESC exhibited the structural and functional characteristics of early CMs [100]. The organization of this differentiation system could have a major impact on the research of early human cardiac differentiation for studies such as functional genomics, pharmacological testing, and cell treatment. Another common 3D method of differentiation is using scaffolds (Figure 2C), which mimic the 3D architecture of the tissue and present specific features such as optimal adhesion, migration, growth, differentiation, and cell functions [87]. Scaffolds can originate from synthetic polymers such as polylactic acid (PLA) or natural polymers such as collagen [102,104,105]. When combined with 3D bio-printing, it is possible, for example, to recreate functional vascularized and perfusable patches that match the immunological, cellular, biochemical, and anatomical properties of the cell donor [106].

Some protocols also combine the use of bioengineering with force to increase cell aggregation and create spherical cardiac microtissues even if they are not able to self-organize or recapitulate the structure and dynamics of the developing heart accurately [107,108,109,110]. When attempting to reproduce the in vivo condition, the use of electrical and mechanical stimuli allows for a better result towards disease modeling [111,112,113]. Many studies show that a highly structured heart shape and cell maturation were induced in cells by electrical stimulation, and when compared to controls without stimulation, there is greater conduction velocity, increased heart-maturation-related gene expression, and electrical signal propagation similar to the in vivo circumstance [114,115]. Similarly, mechanical stimulation increased the CM area, elongation, and sarcomere length and improved calcium handling and the mRNA expression of maturation markers [116]. A specific study was performed where a human tissue-engineered model using cardiac-tissue-engineering methods and cardiomyocytes derived from human iPSC-CMs was developed by encapsulating them in a hydrogel scaffold composed of type I collagen and fibrinogen [117]. This demonstrated the applicability of a human tissue model for studying key aspects of ischemia reperfusion injury and the potential for improving the translation of therapeutic strategies [117].

The last type of 3D cellular culture system is organoids (Figure 2C). Organoids are self-organizing 3D multi-cell structures that can recapitulate organ properties and structure to some extent [61,118,119,120], allowing them to be used for disease modeling. Organoids can be generated from either pluripotent stem cells or progenitor cells. By using primary tissue-derived organoid cultures isolated from patients, it is possible to surpass a general challenge for disease modeling in iPSC-derived tissues, which is the failure to differentiate into mature adult tissue [66].

Self-organizing organoid models can be classified considering four main aspects: (i) a 3D structure containing cells that establish the identity of the modeled organ; (ii) the presence of multiple cell types corresponding to the ones present in the organ that they represent; (iii) the organoid exhibiting a feature based on the function of the organ of interest; and (iv) self-organization according to the same intrinsic method of the organ [58,121,122].

These systems can be maintained in culture for long periods of time [99]; however, oxygen and nutrients are not evenly distributed within the organoid by diffusion, culminating in necrosis of the tissue. This can be overcome by inducing vascularization of the organoid by cellular, genetic, and even bioengineering-based approaches, such as the introduction of artificial microfluidics systems that enhance tissue viability in deeper structures within the organoid [121,123,124,125]. On the other hand, real-time imaging of organoids may be difficult due to tissue thickness, although fluorescent or bioluminescent reporters have been used to improve imaging possibilities [61]. Another limitation of the organoids is the fact that they present a high level of heterogeneity since they have a limited reproducibility [88]. A significant advancement was carried out when researchers developed a method to generate self-assembling hHOs using iPSCs for the modeling of cardiac development and chronic heart disease (CHD) [126]. The hHO were used to model pregestational diabetes (PGD), as it is one of the most prominent factors contributing to CHD. Together, the data point to significant molecular and metabolic changes between normal and diabetic hHOs that are consistent with earlier research on PGD [126]. However, the development of hHO models for cardiovascular disease research has made only small progress and falls behind that of other organs (e.g., kidney, colon, intestine, and brain) despite the importance of comprehending human CVDs.

Although, in the past decade, drastic advancements in studies and protocols for pluripotent stem cell differentiation into cardiac cells have been made, many challenges remain. Differences in individual ESC and iPSC lines, the initial state of pluripotency, epigenetic status (determined by the tissue of origin, in the case of iPSCs), genomic instability of iPSC lines containing epigenetic memory, unusual methylation patterns, mutations generated during the reprogramming procedure and culturing time, line-to-line variability in the yield and purity of CMs, reproducibility and complexity of protocols, and intrinsic differences in endogenous growth factor production between individual lines can affect differentiation efficiency [70,74,127,128,129,130,131,132,133]. In addition, many growth factors used in the differentiation protocols are expensive, have a short half-life, do not readily diffuse into complex multicellular systems, and exhibit lot-to-lot variation in their bioactivity [74]. Thus, important further improvements of pluripotent stem-cell-derived CMs and 3D models in terms of maturity, cell specificity, and standardization of the culturing methods are needed to allow a more comprehensive understanding of the molecular basis of cardiovascular diseases and for the future development of personalized and tailored medicine approaches. A compilation of the main advantages and disadvantages of 2D and 3D models is given in Table 1.

## 4. Human Stem Cells for Toxicity Studies

Toxicity studies investigate the safety profile of a candidate compound such as chemical compounds (e.g., pesticides, food additives, and contaminants), biomaterials, drug delivery systems (such as nanoparticles), and pharmaceutical drugs. Moreover, regarding the search for new and more efficient therapies, toxicological screening is essential not only in drug development but also in repurposing existing drugs for new uses to assess specific adverse events including cardiotoxicity, hepatotoxicity, neurotoxicity, and teratogenicity [135,136,137] since unexpected adverse effects of drugs are a major reason for the termination or withdrawal of drugs [138,139].

The methods for predicting toxicity employ in vitro and in vivo models. However, there are several drawbacks associated with each one. For instance, although in vitro models are especially convenient in predictive toxicology, as they can greatly reduce assay costs and animal usage while identifying those chemicals that may require further in vivo evaluation, these are not as effective as in vivo models to study absorption, distribution, metabolism, and elimination (ADME) since a whole-body organism is generally needed [140]. On the other hand, one disadvantage of in vivo animal models and some in vitro rodent-based assays is the lack of extrapolation to humans, owing to interspecies differences. Due to these limitations, several assays have been developed based on human pluripotent stem cells [141,142] (Figure 2D).

### 4.1. Sources and Types of Human Stem Cells Used in Toxicity Studies

Human stem cells, including ESCs and ASCs, were initially used in toxicity studies and viewed as highly relevant resources to complement in vivo studies. Moreover, the developed assays using human stem cells have shown predictivity, reliability, and reproducibility, conferring them several advantages over other models [143,144,145,146,147].

As discussed before, although ESCs have a wide differentiation potential, their use raises important ethical and regulatory issues limiting their use. Conversely, ASCs have a more limited self-renewal and restricted differentiation potential and are not numerous in the human body [136,148,149]. Nevertheless, these disadvantages have not precluded the development of feasible toxicologic assays. For example, human MSCs isolated from the umbilical cord and human adipose-derived MSCs were presented as novel platforms for the evaluation of the cytotoxicity of pharmaceutical drugs [150,151]. Furthermore, standardized procedures for the evaluation of acute toxicity of nanoparticles were developed and applied using human bone marrow MSCs [152,153].

More recently, the generation of iPSCs and their potential to differentiate into specific somatic cells has led to the development of different approaches. Indeed, iPSCs from normal, diseased, and, importantly, patient-specific donors are being used to generate neurons, hepatocytes, and CMs, among others, for toxicity prediction in different body cells. In developmental toxicology, pluripotent stem cells (ESCs and iPSCs) are used to form EBs that recapitulate events involved with early embryogenesis, such as germ layer formation. They can also be efficiently differentiated into functional CMs both in 2D and 3D models, being suitable for cardiac developmental/teratogenicity and cardiotoxicity studies [154].

### 4.2. Human Stem Cells in Developmental Cardiotoxicity

To prevent congenital malformations due to maternal exposure to drugs and other chemical compounds, safety regulations require a thorough pre-clinical developmental toxicity evaluation. Alternative non-mammalian in vivo, ex vivo, and in vitro assays have been developed to assist in the understanding of the mechanism(s) of toxicity (https://database.ich.org/sites/default/files/S5-R3_Step4_Guideline_2020_0218_1.pdf, accessed on 7 April 2023. Pluripotent stem cells can differentiate into tissue derivatives of all three germ layers in vitro and mimic the embryo development, allowing study on the impact of a compound on mesoderm induction [147,155,156]. Moreover, these cells can further differentiate, enabling the study of cardiac induction, followed by differentiation into CMs, which provides a convenient tool for exploring the molecular and cellular events involved in developmental cardiotoxicity [147,157].

Despite being rapid, simple, and sensitive, the former rodent cell-based assay may not adequately predict human risk in certain instances. Due to this limitation, some assays were developed using human ESCs, and the effects of known toxic compounds on cardiac development were further studied (Table 2).

In one such study, human ESCs were exposed to cyclophosphamide at different stages of differentiation to test whether it induces developmental and cellular toxicity in the human embryo. The authors found that a high concentration of cyclophosphamide could inhibit cardiac differentiation. In addition, cardiac precursors were more sensitive to cyclophosphamide in non-cytotoxic concentrations than late-mature CMs [156]. In another study, trichloroethylene (TCE), a ubiquitous compound in our living environment previously reported to cause congenital heart disease in humans due to prenatal exposure, was studied using human ESCs and ESC-derived CMs. It was found that exposure to low doses of TCE induced significant cardiac differentiation inhibition, which was associated with decreased beating foci. Moreover, the involvement of Ca^2+^ channels in TCE cardiotoxicity, previously reported using animal models, was confirmed, and a novel inhibitory effect of TCE on the differentiation of cardiac progenitors to CMs was discovered [161]. Another study also using a human ESC-CM differentiation model unveiled the underlying mechanism of dioxin and dioxin-related polychlorinated biphenyls, which are potent toxic compounds related to developmental heart defects and congenital heart diseases for which different animal models show distinct susceptibility and phenotypes after exposure, thus suggesting possible species-specific effects. The authors showed that the treatment of human ESCs with 2,3,7,8-tetrachlorodibenzo-p-dioxin (TCDD) at the ESC stage inhibited CM differentiation by promoting aryl hydrocarbon receptor (AHR) binding and repression of key mesoderm genes [157].

In more recent studies, cardiac organoids were also prepared to complement developmental studies. For example, using a human ESC-derived 2D/3D differentiation model system and cardiac organoids, low-dose exposure to cadmium (Cd) was shown to suppress mesoderm formation by affecting the expression of important markers (e.g., HAND1, SNAI2, and HOPX) and cardiac induction through the deregulation of genes such as NKX2-5, GATA4, and TNNT2, and inhibited cardiac differentiation was found by a decreased contractility both in the 2D model and cardiac organoids [178].

The availability of iPSCs led to the development of a variety of embryotoxicity assays that were proven to be more sensitive and biologically relevant than mouse ESC-based studies and without the ethical concerns of using human ESCs (Table 2) [162,175]. Moreover, the viability and differentiation assays previously developed for ESCs were promptly adapted on iPSC-based cardiac developmental and cardiotoxicity assays. In addition to the studies mentioned before, cardiac developmental toxicological effects of several other pharmacologic compounds and already known teratogenic drugs, including arsenic trioxide [160], isotretinoin [158], thalidomide, 5-fluorouracil, and valproate, were demonstrated using human iPSCs [162,176]. One interesting study by Karhu and coworkers showed that the cell types selected for toxicity screening have a major effect on the results. Through a comparative study, it was reported that human stem cells represent the most sensitive screening model to be used in in vitro cardiac developmental toxicology studies [177].

### 4.3. The Use of Human Stem Cell-Derived Cardiomyocytes in Cardiotoxicity Studies

Drug-induced cardiotoxicity is a major problem in the process of drug development and may lead to unexpected life-threatening situations and high costs [179,180]. Therefore, we may assume that animal models and cell lines used in cardiotoxicity studies lack sufficient predictability. On the contrary, CMs derived from human pluripotent stem cells have proven to represent a valuable in vitro model for preclinical cardiotoxicity screenings [144,180,181,182,183]. Thus, alternative assays using iPSC-CMs are increasingly being employed for regulatory decision making [184]. In this context, various protocols have been developed to generate specifically atrial-, ventricular-, and nodal-like CM subtypes from pluripotent stem cells [154,185]. In addition, CM functional readouts were set and included Ca^2+^ and voltage levels, sarcomere contraction forces and organization, ion channel activation, apoptosis, and mitochondrial damage. Some cellular mechanisms have been also associated with cardiotoxicity, such as genotoxicity, oxidative stress, apoptosis, and lipid peroxidization [181,186,187,188,189,190].

iPSCs have been explored by many research groups to identify compounds that are toxic or pose cardiac side effects, such as bisphenol A [167] and ethanol [168]. One relatively frequent side effect of drugs is the inward-rectifier potassium channel (IKr) block and QT interval prolongation, leading to fatal ventricular arrhythmia such as Torsade de Pointes (TdP). The evaluation of the ability of a drug to cause this effect has traditionally been performed by using immortal cell lines expressing the IKr channel, but CMs derived from iPSCs were shown to be more physiologically relevant in this setting [180]. In addition, the increase in atrial fibrillation seen in B-cell cancer patients taking Ibrutinib was confirmed to be due to atrial-specific toxicity in ESC-derived CMs [164]. Moreover, some drugs reduce left ventricular contractile function. This effect is especially represented in anti-cancer drugs, which improve cancer survival but lead often to cardiomyopathies [191]. For example, ponatinib, a tyrosine kinase inhibitor, doxorubicin, and trastuzumab presented cardiotoxic effects in iPSC-CMs [165,171,173]. Importantly, these studies using iPSC-CMs shed light on the molecular mechanisms of toxicity (Table 2). For example, ponatinib exposure was shown to lead to the inhibition of pro-survival signaling pathways, actin cytoskeleton damage, mitochondrial stress, cell death, troponin secretion, and disruption of cardiac cell beating [171]. Doxorubicin promoted alterations in beating profiles due to the dysregulation of cellular pathways related to death receptor signaling, ROS production, and calcium signaling [165]. Trastuzumab provoked cardiotoxic effects via ErbB2 inhibition only when ErbB2/4 signaling was active [173].

In addition, in the context of cancer therapy, iPSC-CMs were used to predict the susceptibility to doxorubicin-induced cardiotoxicity, thus identifying cancer patients at high risk for drug-induced cardiotoxicity and the mechanism of such effects [165] (Table 2). Further studies in this context unveiled biomarkers and led to the development of approaches to prevent toxic effects and specifically treat these patients [192,193,194,195,196]. Therefore, the use of iPSC-CMs allowed the development of new therapeutic approaches against doxorubicin-induced cardiotoxicity, furthering the aim of personalized medicine. Indeed, the case of cancer treatment using anthracyclines is just one example of how the generation of iPSC lines and their derivatives allowed the existence of a unlimited source for the generation of both patient- and disease-specific test systems [196,197] to develop individual-specific predictions of adverse event risk and adapt the clinical approach to a specific patient.

While some technical issues still exist for the widespread use and implementation of human stem-cell-based assays into drug discovery and toxicity testing, these have the irrefutable potential for safer, more customized, and accurate risk assessment and are associated with reduced economical and ethical costs. Moreover, as illustrated by the previously described studies, these assays also have the advantage of providing insights into the molecular basis of (cardio)toxicity [198].

## 5. Stem-Cell-Based Therapies for CDVs

Several factors must be considered when designing a clinical trial to obtain optimal results with stem-cell-based therapy: the type of cells to be used (addressed earlier in this review), the cell dose, the route of injection/infusion, and the time window of the treatment, which will be discussed below.

### 5.1. Route of Cell Administration

Clinicians have been working on a way to efficiently deliver stem cell-based medicinal products without compromising their therapeutic potential. Transendocardial stem cell injection (TESI) releases the cells from a catheter directly into the myocardium through the endocardium (Figure 2E). It is a minimally invasive, safe, and feasible method. Still, there is a low risk of myocardial perforation and arrhythmia induction. TESI was shown to induce a reduction of the infarct size while improving cardiac function both in animal models and clinical trials. According to Kanelidis’s meta-analysis, the TESI method presents the best results, with the benefits outweighing the risks, in animal models of MI and left ventricular ejection fraction (LVEF) [199]. In the intramyocardial injection, the cells are injected directly into the myocardium (Figure 2E) through a thoracotomy, allowing a precise and accurate epicardial approach method. However, it is a very invasive surgical approach, which entails a high risk of post-operative complications, morbidity, and mortality. This technique is specially used to investigate chronic ischemic cardiomyopathy (ICM) clinical trials because it can be performed during an open-heart surgery [199]. The intravenous infusion (IV) is the least invasive method, in which the cells reach the injury site via the bloodstream (Figure 2E). However, because they are in circulation, cells tend to not be retained and implanted in the infarcted area, migrating to other organs such as the lungs, kidney, or liver or even being eliminated, thus decreasing the efficiency of the therapy [24,199]. The intracoronary infusion (IC) method allows the cells to be infused from the coronary artery or the cardiac vein to the infarcted myocardium (Figure 2E) [24]. The main advantage of this method is that it allows a high number of cells to reach the ischemic area without damaging the myocardium. However, the dose of cells infused is a limiting factor since if a large dose is injected, it may cause artery occlusion, causing a new MI [31,199].

### 5.2. Preconditioning Stem Cells to Obtain Better Clinical Outcomes

There are several factors limiting the effectiveness of stem cell therapy, but the poor outcomes are mainly due to the limited retention and survival of the cells in the affected area.

It is known that after an MI episode, the infarcted area is highly hypoxic, constituting a hostile environment for the transplanted cells that are normally cultured in normoxia (20% O_2_), thus causing a low level of engraftment. It was proven that culturing MSCs in hypoxic conditions (5% O_2_) extends cellular lifespan and proliferation and attenuates their differentiation [200,201]. Growth factors such as IGF-1, HGF, and bFGF have also been used to treat stem cells prior transplantation, showing an improvement of cell survival, proliferation, and cellular differentiation and an increased release of paracrine factors in vitro [201]. Preconditioning adipose tissue-derived MSCs with n-butylidenephthalide (BP) increased stem cells survival, induced their differentiation into cardiomyocytes via STAT3 pathway, and promoted M2 macrophage migration into the injured tissue, attenuating myocardial fibrosis [202]. Additionally, Bortolotti and colleagues showed that treating MSCs with cardiotrophin-1 promotes cell grafting, improve cardiac function, and decrease scar tissue [203].

### 5.3. Timing Matters

In most pathologies, the time interval in which cells are transplanted to the patient after an event such as heart failure or acute MI is critical to optimize the efficiency of the therapy. Xu’s team developed a meta-analysis of randomized controlled trials that occurred between 2000 and 2017 to understand the best timing for autologous BMMNCs transplantation in acute MI [204]. Their results showed an improvement in cardiac function when cell transplantation occurred between 3 and 7 days after the event, resulting in a significant increase in LVEF and prevention of ventricular remodeling. When the transplantation occurred within 24 h after an acute MI, the infarction area was in a high inflammation state, resulting in early differentiation of BMMNCs. On the other hand, after a 7-day window, scar tissue was formed, and there was an accumulation of fibroblasts at the site, which negatively impacted BMMNCs differentiation [204].

### 5.4. From Benchside to Bedside with Stem Cell-Based Therapies

Performing a search on the *Clinicaltrial.org* database with the words “*cardiac disease*”, “*myocardial*”, and “*stem cell*”, 393 clinical trial records were obtained. From these, 185 clinical trials were completed, and there were 16 published results involving the direct use of stem cells. A summary of these trials can be found in Table 3.

Amongst the cardiac diseases studied in these clinical trials, heart failure [205,206,207,208] and left ventricle dysfunction [33,205,209,210,211,212,213] were the primary targets, and the participants were mostly adults aged between 50 and 70 years [33,205,206,207,208,209,210,211,212,214,215,216,217]. The cell types used in these clinical trials were BMMNCs [33,205,206,210,214,216,218], bone marrow MSCs [207,208,212,215,219], bone marrow total nucleated cells [211], CD34^+^ cells [217], cardiac stem cells [211], cardiosphere-derived cells [220,221,222], and c-kit-positive cardiac cells [207,208], while autologous bone marrow MSCs were the most-used cell type. We also observed that, in general, the cell dose used was in a range between 1 × 10^7^ and 1 × 10^8^ cells/patient, and TESI was the most-used method to deliver the cells [33,205,206,207,208,214,216].

From the 16 trials described in Table 3, five used BMMNCs (NCT00203203, NCT00684021, NCT00684060, NCT00768066, and NCT00824005). The clinical trial with the reference NCT00203203 aimed to administer 3 × 10^7^ autologous BMMNC via TESI in patients with chronic ischemic heart failure. The results showed that this therapy was safe, presenting similar adverse events in both experimental groups, significant improvement in the quality of life of treated patients, and an improvement of the myocardial volume oxygen (MVO_2_) level in younger patients [206]. In the clinical trial with the reference NCT00684021, in which the objective was to determine the best timing for BMMNCs administration in patients with left ventricular dysfunction, 1.5 × 10^8^ BMCs or the placebo were administered at day 3 or day 7 after percutaneous coronary intervention. In this study, no differences between the treated and the placebo groups were observed. The authors suggested the poor outcomes may be due to the release of reactive oxygen species and inflammatory cytokines from circulating inflammatory cells following ST-segment-elevation myocardial infarction (STEMI), which may negatively affect the survival of BMMNCs. The heterogeneity among patients was also cited as a confounding factor. Finally, it was also speculated that bone marrow cells from patients with cardiomyopathies may be compromised due to a lower cytokine production and a lower colony-forming-unit capacity. Thus, the authors considered that, in this type of assay, autologous cells may have a lower regenerative capacity when compared to allogeneic cells from healthy young donors, suggesting an allogenic administration in future trials [210].

The same author participated in another trial (NCT00684060) to determine the effect of late intracoronary delivery of autologous total nucleated bone marrow cells on the recovery of left ventricular function following STEMI and study the influence of timing. Although no difference between the treated and control groups in both timepoints was observed, this trial demonstrated its safety and feasibility (Table 3). Here, the administration occurred during a transition between the inflammatory phase and the proliferative phase. It is known that during the proliferative phase, there is an increase in neovascularization and extracellular matrix formation in the injured region. Furthermore, in the first days after MI, there is a major release of progenitor cells from the bone marrow to the affected cardiac region. Thus, it is possible that at the time of bone marrow aspiration, there may be low concentrations of progenitor cells, and consequently, the quality of the collected cells may be affected. The cell quality is also negatively affected by some additional factors such as the advanced age of the participants and other cardiovascular risk conditions. Overall, these factors could negatively affect the beneficial effects of BMCs, contributing to poor outcomes [209].

The clinical trial with reference NCT00768066 intended to prove the safety of 10 TESI with autologous MSCs and BMMNC in patients with ischemic cardiomyopathy (ICM) or MI. In all the patients, the TESI procedure was successful; the therapies were safe, with no difference of serious adverse effects between the groups, and no ectopic tissue formation was detected. However, only MSCs exerted regenerative and anti-fibrotic effects, with improvement of functional capacity and quality of life [214].

**Table 3:** Completed clinical trials related to cardiac diseases and stem cells.

Clinical Trial Number	Type and Status	Condition(s)	Cell Type(s)	Population	Results	References
NCT00203203	Randomized, phase I, single-blind, placebo-controlled trial; proportion 2:1 (placebo); status: complete	Chronic ischemic HF and no option for revascularization	TESI of autologous BMMNC (3 × 10^7^ cells)	30 participants; age: 56.3 ± 8.6 years old (control group), 60.5 ± 6.4 years old (treatment group); sex: all	Cell therapy was safe and with similar adverse events in both groups, significant improvement in quality of life of treated patients, and increase of the MVO_2_ level in younger patients	[206]
NCT00587990	Randomized, phases I and II, prospective study; status: terminated due to difficulties to recruit	Chronic ischemic left ventricular dysfunction secondary to MI	DI of autologous BM-MSC	6 participants; age: 54.9 ± 4.2 years old; sex: male	MSC acts in the injection site, with improvement of LV function, reduction in scar size, and improvement of perfusion and contractility	[223]
NCT00684021	Randomized, phase II, double-blind, 2 × 2 factorial, placebo-controlled trial; status: complete	LV dysfunction	IC of autologous BMMNC (1.5 × 10^8^ cells)	120 participants; age: 56.9 ± 10.9 years old; sex: all	After reperfusion of STEMI, LV function improved without influence of BMMNC	[210]
NCT00684060	Randomized, phase II, double-blind, placebo-controlled trial; proportion 2:1 (placebo); status: complete	Significant LV dysfunction	IC of autologous total nucleated cells from bone marrow (1.5 × 10^8^ cells)	87 participants; age: 57.6 years old (treated group), 54.6 years old (placebo group); sex: all	Safe and feasible, with no significant change in LV function at 6 months	[209]
NCT00768066	Randomized, phases I and II, double-blind, placebo-controlled trial; proportion 2:1; status: complete	ICH; Chronic MI	10 TESI in LV of autologous MSC or of total BMMNC	65 participants; Mean age: 59.9 years old; Sex: all	Safe, with no serious adverse effects in any of the groups; MSC exerts regenerative and anti-fibrotic effects, with improvement of functional capacity and quality of life	[214]
NCT00824005	Randomized, phase II, double-blind, placebo-controlled trial; proportion 2:1; status: complete	CAD, LV dysfunction, limiting heart failure, angina	TESI of autologous BMMNCs (1 × 10^8^ cells)	92 participants; mean age: 63 years old; sex: all	The injection of BMMNCs did not have a positive effect on LVESV and MVO_2_; reversibility was detected on SPECT	[205]
NCT01087996	Randomized, phases I and II, pilot study; proportion 1:1; Status: complete	LV dysfunction	TESI of allogeneic or autologous BM-MSCs (2 × 10^7^ or 1 × 10^8^ or 2 × 10^8^ cells)	30 participants; mean age: 62.8 years old (allogeneic group), 63.7 years (autologous group); sex: all	Low immune reaction in patients who received allogenic BM-MSCs, with improvement of functional capacity, quality of life, and ventricular remodeling in both groups	[33]
NCT01273857	Non-randomized, phase I, prospective controlled exploratory study; status: complete	HLHS and single-ventricle physiology	IC of autologous CDC (3 × 10^5^ cells/kg)	7 participants; age ≤ 6 years old; sex: all	Safe and feasible, improvement of RVEF, no severe adverse effects, and low immune reaction in patients who received allogeneic	[222]
NCT01392625	Randomized, phases I and II, proportion 1:1; status: complete	Chronic NIDCM	Autologous vs. allogeneic BM-MSC	37 participants; age: 55.8 ± 11.2 years old; sex: all	Safe and feasible in both cell types, and allogeneic MSC was more effective than autologous MSC	[215]
NCT01508910	Randomized, double-blind, multicenter trial; proportion 1:1:2; status: complete	Class III or IV angina, who experienced ischemia on stress testing	DI of autologous CD34+ cells	112 participants; median age: 64 years	Safe and yielded improvement of exercise tolerance and decreased angina	[217]
NCT01829750	Randomized, phase II, controlled study; proportion 1:1; status: complete	Single-ventricle physiology	IC of endogenous CDC	41 participants; age: 2.7 ± 1.5 years old (control group), 2.5 ± 1.1 years old (CDC-treated group); sex: all	Significant improvement of cardiac function and reduction of ventricular volume and fibrosis and heart failure status	[220]
NCT02013674	Randomized, phase II, blinded; proportion 1:1; status: complete	Chronic ischemic left ventricular dysfunction secondary to MI	TESI of allogeneic BM-MSCs (2 × 10^7^ cells vs. 1 × 10^8^ cells)	30 participants; age: 66.2 ± 10.7 years old; sex: all	Safe and feasible, with reduction of infarct scar size and improvement of cardiac function or both treatment groups, and the group with 1 × 10^8^ cells had increased ejection fraction	[216]
NCT02439398	Randomized, phases I and II, multicenter, double-blind, placebo-controlled trial; proportion 2:1; status: complete	STEMI and LV dysfunction	IC of allogeneic CSC (1, 2 or 3.5 × 10^7^ cells)	66 participants; mean age: 56 ± 12 years old (treated group), 55 ± 8 years old (control group); sex: all	Safe and feasible. No significant differences in LV size or patient quality of life, between the treated and the control groups	[211]
NCT02467387	Randomized, phase II, single-blind, placebo-controlled trial; proportion 1:1; status: complete	Non-ischemic cardiomyopathy	IV of ischemia-tolerant allogeneic BMMNC (1.5 × 10^6^ cells/kg)	22 participants; mean age: 47.3 years old; sex: all	Safe, with immunomodulatory effects promoted by it-MSCs and improvements in functional capacity and quality of life	[218]
NCT02501811	Randomized, phase II, double-blind, placebo-controlled trial; proportion 1:1:1:1; status: complete	Chronic ischemic HF	TESI of autologous BM-MSC and CPCs (c-kit+ cells), alone or in combination	125 participants; mean age: 62.5 years old; sex: all	Safe and feasible, with improvement of MACE in patients treated with CPCs and quality of life in patients treated with BM-MSC and improvement of MACE and quality of life in patients treated with CPC and MSC	[207,208]
NCT03129568	Non-randomized, phase I, open-labelled prospective study; status: complete	Dilated cardiomyopathy	IC of autologous CDCs (3 × 10^5^ cells/kg)	5 participants; mean age: 11.4 ± 6.7 years old; sex: all	Safe and feasible	[221]

BMMNC, bone marrow mononuclear cells; BM-MSC, bone marrow mesenchymal stem cells; CAD, coronary artery disease; CDC, cardiosphere-derived cells; CSC, cardiac stem cells; DI, intramyocardial injection; HF, heart failure; HLHS, hypoplastic left heart syndrome; hMSC, human mesenchymal stem cell; IC, intracoronary infusion; ICM: ischemic cardiomyopathy; it-MSC, ischemia-tolerant mesenchymal stem cells; LV, left ventricle; LVESV, LV end systolic volume; MACE, major adverse cardiac events; MI, myocardial infarction; MSC, mesenchymal stem cells; MVO_2_, maximal oxygen consumption; NIDCM, non-ischemic dilated cardiomyopathy; RVEF, right ventricular ejection fraction; SPECT, single-photon emission tomography; STEMI, ST-segment-elevation myocardial infarction; TESI: transendocardial stem cell injection.

Perin and colleagues conducted a clinical trial (NCT00824005) to evaluate the safety and efficacy of TESI to administer BMMNCs in patients with CHD and left ventricular dysfunction. Their results showed no differences between the transendocardial injection of BMMNCs and the placebo in left ventricular end systolic volume, maximal oxygen consumption, or reversibility on single-photon emission tomography [205]. Although the use of BMMNCs in these trials was shown to be safe, they differed in efficacy regarding cardiac function. Considering that all of them used the TESI route to administer the cells, it can be suggested that the lack of efficacy is not entirely due to this route of administration. It would be interesting to know the severity of the pathology among the participants of the different trials as well as their ages to better understand the variability of the results.

In addition to the clinical trials using BMMNCs, six other clinical trials used BM-MSCs: NCT00587990, NCT01087996, NCT01392625, NCT02013674, NCT02467387, and NCT02501811. In general, all these trials showed positive results, with the improvement of cardiac function and quality of life, with no significant adverse effects regardless of the origin of the MSCs (autologous vs. allogeneic).

The clinical trial with the reference NCT00587990 was developed in patients with chronic ischemic left ventricular dysfunction secondary to MI, where the study tested intramyocardial injections of autologous MSCs in the segments of the myocardium without revascularization. The results showed that MSCs act in the injection site, reducing scar size but also showing improvement of LV function, perfusion, and contractility [212].

Hare and colleagues (NCT01087996) conducted a clinical trial with the objective of comparing the safety and efficacy between autologous and allogeneic BM-MSC in patients with left ventricle dysfunction. They observed a low immune response in patients who received allogeneic BM-MSC and improvement of cardiac function, quality of life, and ventricular remodeling in both treated groups [33]. The same team led another clinical trial (NCT01392625) with similar objectives in chronic non-ischemic dilated cardiomyopathy. They obtained good results in patients treated with allogeneic BM-MSCs [215].

In the clinical trial with the reference NCT02013674, TESI was tested to administer two different doses (2 × 10^7^ cells vs. 1 × 10^8^ cells) of allogenic BM-MSCs in patients with ICM. In both groups, the trial showed to be safe and feasible. Reduction of infarct scar size and improvement of cardiac function was also observed. In addition, the group that received the highest cells dose (1 × 10^8^ cells) presented an increase in ejection fraction [216].

Butler and his team performed the clinical trial with the reference NCT02467387 with the objective to test the safety and efficacy of ischemia-tolerant MSC after intravenous administration in patients with non-ischemic cardiomyopathy. The results demonstrated that this therapy is safe, and ischemia-tolerant MSC promotes a modulatory effect on the inflammatory system, with patients presenting improvements in functional capacity and quality of life [218].

The clinical trial with reference NCT02501811 differed from the previous trials because it tested the efficacy and safety of using BM-MSCs alone or in combination with c-kit^+^ cardiac cells in patients with heart failure caused by ischemic cardiomyopathy. In the experimental group treated with CPC, the study observed an improvement of major adverse cardiovascular events (MACE). In the group treated with BM-MSCs, an improvement of quality of life was observed. When treated in combination with both cell types, the participants showed an improvement in MACE and an increase in quality of life [208]. This trial also demonstrated that it is safe to use MSCs in combination with other cell types.

Cardiosphere-derived cells (CDCs) were used in three clinical trials (NCT01273857, NCT01829750, and NCT03129568) for the treatment of pediatric pathologies.

Ishigami and colleagues directed a phase I and a phase II clinical trial (NCT01273857 and NCT01829750, respectively) aiming to test the safety and feasibility of intracoronary delivery of CDCs in children with hypoplastic left heart syndrome. The results demonstrated that the use of CDCs is safe and feasible, with no severe adverse effects. They observed an improvement of right ventricular ejection fraction and a significant reduction of right ventricular volume, fibrosis, and heart-failure status in CDC-treated patients [220,222].

The clinical trial with the reference NCT03129568 aimed at determining the safety and feasibility of intracoronary infusion of CDCs in patients with dilated cardiomyopathy. The results demonstrated that CDC therapy is safe and feasible. However, no significant improvement of LVEF at 6 and 12 months after treatment was observed. This clinical trial involved only five participants due to the low annual incidence of pediatric dilated cardiomyopathy, and for this reason, it was not possible to obtain a control group to better understand the effects of this therapy. Furthermore, there was great heterogenicity among cardiac phenotypes, making it difficult to compare the therapeutic responses among patients after CDC infusion [221].

Only one clinical trial used autologous CD34^+^ cells (NCT01508910) in patients with refractory angina to analyze their ability to decrease symptoms of angina and increase exercise capacity. In this trial, patients were treated with granulocyte colony-stimulating factor (G-CSF) for four days, and on day 5, a peripheral blood sample was taken. CD34^+^ cells were isolated, and the quality of the sample was analyzed. Ninety-six hours after blood collection, the CD34^+^-enriched cell suspension was administered through intramyocardial injection back to the patient. Although CD34^+^ cell therapy was demonstrated to be safe, with improvement of exercise tolerance and decreased angina, the results are only suggestive due to the early termination of the trial [217]. The clinical trial NCT02439398 aimed to establish the safety and feasibility of intracoronary infusion of allogeneic CSCs in patients with STEMI. It demonstrated the safety and feasibility of CSCs-based therapy in patients with low immunogenicity. However, no benefits in infarct size reduction, left ventricular remodeling, or differences in quality of life between the CSCs- and placebo-treated group were observed. The authors concluded that it will be advantageous to increase the number of participants in this study to demonstrate the potential efficacy of this type of cell in the treatment of CVDs. They also considered testing other strategies such as a higher dose of CSCs, several administrations, a combination of various cell types, and testing other delivery routes of administration such as the intramyocardial or intrapericardial routes [211].

The overall data obtained from the completed clinical trials and the advances in cell therapy research led to the design of new clinical trials using different cell types, such as ESCs and iPSCs. There are currently 48 clinical trials active or recruiting participants. Discarding those involving the concomitant use of pharmacological therapy or the ones that are only observational, there are 24 trials, which are described in Table 4, with no published results available yet. Compared to the completed trials discussed above, these new clinical trials differ in some aspects. Although MSCs are still the cell type of election due to, as previously described, their low immunogenic profile and immunomodulatory potential, among others, in the new trials, the cell source is predominantly the umbilical cord tissue instead of the bone marrow. Therefore, clinical trials are using more allogeneic than autologous transplantations. Moreover, there are now four clinical trials using iPSCs and one clinical trial using ESCs. In both cases, these pluripotent stem cells are differentiated in CMs before transplantation into patients.

Most of these clinical trials are focused on adult and older adult participants suffering from heart failure and/or MI. However, other diseases of interest, such as hypoplastic left heart syndrome and congestive heart failure, are starting to emerge. In these new trials, the cell dose established is between 1 × 10^7^ and 1 × 10^8^ cells per patient, which is similar to that demonstrated in the previous clinical trials to be safe and with no earlier major side effects. Regarding the administration route, new trials are using the transendocardial, intramyocardial, intracoronary, and especially the intravenous route. In addition, these studies are also starting to use combined tissue-engineering medicinal products, in which stem cells are inserted in a patch or diluted in a hydrogel.

## 6. Ethical Issues and Other Challenges

Since the successful culture of human ESCs in the late 1990s, high expectations have been raised to translate stem cell research from the bench to the bedside for the treatment of damaged myocardial tissue. Rapid advances in the field have pressed the development of the ethical and regulatory laws and policies that guide stem cell research. The use of human pluripotent stem cells has been the source of some ethical controversy, mainly because it primarily relied on deriving those cells from ESCs obtained from human embryos, including discarded embryos from in vitro fertilization processes and embryos unsuitable for implantation, which ultimately results in the destruction of the embryo [224]. Thus, research in this field has been addressed by legislators and licensed by different statutory regulatory entities that monitor licensed research. However, some constraints related to the funding of research that implies the destruction of human embryos had been circumvented by using existing cell lines generated from ESCs. In 2001, the U.S. government restricted the funding for research on ESCs, and in 2007, the European human Embryonic Stem Cell registry (hESCreg) supported by the European Commission was created from a recommendation of the European Group on Ethics to provide a list showing the stem cell lines available and the legislative environment in each country [225]. To address the variety of legislation across the world, the International Society for Stem Cell Research (ISSCR) became the preeminent global organization dedicated to all aspects of stem cell research. Since its foundation in 2002, and given the continuous scientific advances, the ISSCR created and has been updating its Guidelines for Stem Cell Research and Clinical Translation [223].

As scientific progress has led stem cell research to widen its applications, such as cell-based therapies and drug screening, other sources that generally do not raise controversy have been explored to establish human pluripotent stem cells. As previously discussed, ASCs in mature tissues have been isolated and expanded for tissue renewal and repair but also show some limitations. The differentiation level of these cells, which are more committed to differentiating into specific cell lineages than ESCs, results in low rates of retention and differentiation into the desired cell, such as CMs [24]. Moreover, ASCs are difficult to maintain and represent a very reduced population, impairing their use for therapeutic development. There are also some challenges regarding the donors. Adding to some possible level of discomfort and risk for the donor during cell collection, it is necessary to assure an adequate informed consent procedure, while financial counterparts are currently prohibited. The breakthrough in stem cell research that allowed bypassing previous challenges was the isolation and reprogramming of different types of somatic cells into iPSCs. This technology was well received by the opponents of funding for research with human ESCs, raising no controversy while showing promising potential in cardiac regeneration. Nevertheless, despite the great promise of iPSCs, there are some hurdles to overcome before their therapeutic use. It is important to reset molecular identity, achieve more efficient and faster non-integrative methods for iPSCs generation, and manage their tumorigenic potential [219].

The ISSCR guidelines have followed scientific developments in stem cell science, incorporating iPSC research and applications [226]. More recently, the application of genome editing, embryo models, and organoids pressed the ISSCR guidelines to recommend that unique aspects of human embryo research and associated ethical issues should be evaluated through a specialized oversight process [213]. Although there has been an increase in the number and use of ESCs and iPSCs lines, due to their source, derivation processes, and subsequent handling procedures, most of them are only suitable for research [227]. Nevertheless, rigorous donor screening and the quality of the final product in the generation of clinical-grade human pluripotent stem cells are ensured by the implementation of regulatory policies on current good manufacturing practice (cGMP) [228]. As for other malignancies, stem cell therapy for CVDs still faces some challenges. Effective treatment of heart disease patients with stem cell therapy remains to be determined, namely the type, source, and dose of stem cells; the delivery method; and the mechanisms by which the delivered cells exert their effects. Several other factors can influence the outcomes of stem cell therapy, such as the etiology of the heart disease, comorbidities, the patient’s age, or the need for surgical procedures to deliver the cells [229,230]. Randomized clinical trials using stem cells in cardiovascular medicine differ from the traditional clinical trials and exhibit more scientific and ethical challenges. Among these is the assessment of risks and benefits, including the invasiveness of the intervention, and the inclusion of sham procedures, which are only ethically acceptable if scientific and societal benefits are similar to the physical and psychological risks. Moreover, the specific characteristics of stem cells as well as the novelty and complexity of clinical trials using them entail an underlying uncertainty depending on the type of stem cells or the phase of the trial. These challenges are accompanied by high expectations driven by the inherent interests of all the parties involved [231]. Despite the promising results in some of the trials presented above, multiple studies in the past two decades have shown that neither ESCs nor human ASCs and iPSCs can fully regenerate adult tissues, including cardiac tissue. Thus far, clinical studies have failed to differentiate those cells and functionally integrate them in mature tissues, and there is still a long way to go to achieve a safe and efficient application of stem cells and their derivatives in disease therapy and regenerative medicine [226].

## 7. Perspectives and Conclusions

The development of human stem cells has been a significant milestone in stem cell research, and their medical applications are now flourishing. Indeed, various types of stem cells have been tested, including ESCs, ASCS, and iPSCs.

Stem cells have become a crucial tool for modeling CVDs, as they can recapitulate the endogenous mechanisms of the human organism in vitro and provide important insights into the molecular basis of cardiovascular development, disease progression, and pathogenesis. Since the studies of Takahashi in 2006, iPSCs have become a model of choice. iPSCs can be differentiated into cardiac cell lineages using either 2D in vitro models, which are easy to establish, or 3D models, which provide a more complex cellular organization, structure, and environment, resembling better the natural heart tissue. Both models have advantages and disadvantages for CVD research, and the choice depends on the research question and available resources. Nevertheless, the 2D and 3D models already provide complete cellular systems that can be used for drug screening and to assess cardiotoxicity. The use of human stem cells in toxicity studies also offers several advantages over other models, including predictivity, reliability, and reproducibility, providing a tremendous resource for human material in toxicology research. Thus, these models are complementary to or can even replace in vivo models, which have limitations such as a lack of effectiveness in ADME studies due to interspecies differences. In addition, advances in iPSCs offer the possibility to generate patient-specific cells to devise personalized medicinal treatments.

On the other hand, although ESCs, iPSCs, and ASCs have been tested to treat CVDs, at present, none of these clinical trials has yielded satisfying and sustained results for regeneration of the damaged heart tissues or even for the long-term improvement of the heart conditions. However, apart from the right (stem) cell type to use, the route of cell administration and timing of transplantation for an efficient and safe delivery of stem cells, which are critical factors for a successful intervention, have not been solidly determined. Most trials showed encouraging results, indicating that stem-cell-based therapy was safe and feasible, with improvements in heart function, LVEF, and/or quality of life. However, while most clinical trials showed positive results, the majority have failed to differentiate those cells and functionally integrate them in mature tissues. Survival and engraftment are currently the most important challenge for stem cell therapy, considering that 90% of the injected cells die by apoptosis [232].

Several studies have focused on strategies to optimize stem cell migration through damaged myocardial tissue. Paracrine actions of anti-apoptotic, immunomodulatory, or proangiogenic factors and/or secreted vesicles (such as exosomes) from stem cells have been shown to be responsible for many of the therapeutic benefits observed in stem-cell-based therapies.

Therefore, alternative cell-free strategies, such as the activation of endogenous cardiac cells’ proliferation and/or differentiation, usage of exosomes derived from (stem) cells, and direct reprogramming of somatic cells into cells of the heart, are now being explored. However, the safety concerns with the use of viral vectors in direct reprogramming need to be addressed. Furthermore, the development of new delivery methods based on novel nanotechnology could enhance the efficacy and safety of stem-cell-based therapies.

In conclusion, stem cell research has made significant progress in the field of cardiovascular regenerative medicine over the past few decades. However, there is still a long way to go before stem cell therapy can be safely and effectively applied in disease therapy and regenerative medicine. Therefore, further research is needed to overcome these challenges and realize the full potential of stem cells in cardiovascular medicine.

## Figures and Tables

**Table 1:** Advantages and Disadvantages of Pluripotent Stem Cells derived in vitro models.

Model		Advantages	Disadvantages	References
2D	Monolayer	- User-friendly - Controlled - Reproducible - Cost-effective - Even distribution of nutrients and growth factors - Homogeneity - No purification steps needed	- Fails to recapitulation the complexity of human physiology - Lack of cell–cell and cell–ECM interactions - iPSCs-CM immaturity	[68,72,80,91]
	Co-Culture	- Requires few cells - Presence of multiple cell types - Cell–cell and/or cell–ECM interaction - High range of applications - Easy and rapid - Produces high amount of quality cells	- Fails to recapitulate the complexity of human physiology - iPSCs-CM immaturity	[80,94,95,96]
3D	EBs	- User-friendly - Scalable - Controlled - Improved iPSCs-CMs maturity - Improved recapitulation the complexity of human physiology	- Technical difficulties (e.g., pipetting and medium changes) - Heterogeneity - Can require large volumes - Expensive - iPSCs-CM immaturity - Lack of nutrients in the core of spheroid	[16,66,72,103,105,106]
	Scaffolds	- Three-dimensional architecture of the tissue and present specific features - Model can be improved when combined with external stimuli - Improves the viability of cells - Improved iPSCs-CMs maturity	- Higher costs of bioprinting and bioengineering - More complex protocols	[114,115,116,117,118]
	Organoids	- Higher recapitulation of organ properties and structure - Higher recapitulation of organogenesis - High range of applications - Higher iPSCs-CMs maturity	- Heterogeneity - Uneven distribution of oxygen and nutrients - Difficulties with real-time imaging - Complex, expensive, and long differentiation protocols	[60,63,69,91,124,125,126,127,128,134]

**Table 2:** Cardiotoxicity studies using human pluripotent stem-cell-derived models.

Study Type	Stem Cell Type	Compound(s) Tested	Main Effects/Mechanism	Reference
Developmental Cardiotoxicity	ESCs	Cadmium	Cardiac differentiation inhibition through repression of key mesoderm and cardiac induction genes	[158]
Developmental Cardiotoxicity	ESCs	Cyclophosphamide	Cardiac differentiation inhibition	[159]
Developmental Cardiotoxicity	ESCs	Perfluorooctane sulfonate (PFOS)	Cardiomyocyte toxicity through induction of lysosomal dysfunction after blocking autophagic flux and resulting in mitochondrial dysfunction	[160]
Developmental Cardiotoxicity	ESCs	2,3,7,8-tetrachlorodibenzo-p-dioxin (TCDD)	Cardiac differentiation inhibition through repression of key mesoderm genes	[161]
Developmental Cardiotoxicity	ESCs	Trichloroethylene (TCE)	Cardiac differentiation inhibition involving the calcium turnover network	[162]
Developmental Cardiotoxicity	ESCs iPSCs	Isotretinoin (13-cis-retinoic acid)	Cardiac differentiation inhibition through dysregulation of genes related to mesoderm specification	[163]
Developmental Cardiotoxicity	iPSCs	Arsenic trioxide (ATO)	Affected proliferation, cellular viability, and cardiac differentiation inhibition	[164]
Developmental Cardiotoxicity	iPSCs	Thalidomide, valproate, aminopterin, methotrexate, 5-FU, RA, tetracycline, lithium, phenytoin, and warfarin.	Cardiac differentiation inhibition	[165]
Cardiotoxicity	ESCs	Cadmium	Reduced cell viability, increased apoptosis, cardiac sarcomeric disorganization, elevated reactive oxygen species, altered action potential profile, and cardiac arrhythmias	[166]
Cardiotoxicity	ESCs	Ibrutinib (IB)	Atrial cell-specific effects on hPSC-CMs, including a decrease in the action potential and an increase in the calcium transient duration	[167]
Cardiotoxicity	iPSCs	Anthracyclines (idarubicin, doxorubicin, epirubicin, and daunorubicin)	Alterations in beating profiles due to the dysregulation of cellular pathways related to death receptor signaling, ROS production, and calcium signaling in hiPSC-CMs	[168]
Cardiotoxicity	iPSCs	Azithromycin (AZM)	Excessive autophagosome formation and accumulation in hiPSC-CM, leading to vacuole formation, sarcomeric damage, and cardiomyocyte death	[169]
Cardiotoxicity	iPSCs	Bisphenol A	Reduced field potential and inhibition of ion channels and contraction in hiPSC-CMs	[170]
Cardiotoxicity	iPSCs	Ethanol	Irregular calcium transients and contractility in hiPSC-CMs	[171]
Cardiotoxicity	iPSCs	Melphalan	Oxidative stress, Ca^2+^ handling defects, and dysfunctional contractility and death in hiPSC-CMs	[172]
Cardiotoxicity	iPSCs	Nefazodone	Inhibition of various voltage-gated ion channel currents, including IKr, IKs, INa, and ICa, leading to arrhythmogenicity in hiPSC-CM	[173]
Cardiotoxicity	iPSCs	Ponatinib	Inhibition of pro-survival signaling pathways, structural cardiac alterations, and disruption of cardiac cell beating in hiPSC-CMs	[174]
Cardiotoxicity	iPSCs	Tebuconazole (TEB)	Alteration of cardiomyocyte’s electro-contractile properties in hiPSC-CMs	[175]
Cardiotoxicity	iPSCs	Trastuzumab	Cardiotoxic effects via ErbB2 inhibition only when ErbB2/4 signaling is active	[176]
Cardiotoxicity	iPSCs	Yohimbine	Inhibition of the frequency of spontaneous action potentials and prolonged action potential duration in hiPSC-CMs	[177]

5-FU, 5-fluorouracil; ESCs, embryonic stem cells; hPSC-CMs, human pluripotent stem-cell-derived cardiomyocytes; hiPSC-CMs, human induced pluripotent stem-cell-derived cardiomyocytes; iPSCs, induced pluripotent stem cells; RA, retinoic acid.

**Table 4:** Active and recruiting clinical trials related to cardiac diseases and stem cells.

Clinical Trial Number	Type	Condition(s)	Cell Type	Population
NCT04776239	Randomized, double-blind	DM and ischemic heart disease	IV of allogeneic MSC (1 × 10^8^ cells)	30 participants; age: ≥18 years old; sex: all
NCT05147766	Single group assignment, open-label	Congestive heart failure and angina	IV of cultured allogeneic UC-MSCs (1 × 10^8^ cells)	20 participants; age: child, adult, older adult; sex: all
NCT04996966	Randomized, parallel assignment	Ischemic heart disease, lung injury, and non-cardiac surgery	IV of allogeneic UC-MSC (1 × 10^6^ cells/kg)	20 participants; age: 60–80 years old sex: all
NCT04728906	Single group assignment	MI and heart diseases	Heart patch seeded with autologous CM and AECs	10 participants; age: 40–60 years old; sex: all
NCT02781922	Randomized, parallel assignment	HLHS and single ventricle	IC of autologous CSC (3 × 10^5^ cells/kg).	40 participants; age: 0–6 years old; sex: all
NCT04907526	Non-randomized, parallel assignment	Single right-ventricle-dependent congenital heart disease	DI of autologous mononuclear cells (3 × 10^6^ cells/kg).	30 participants; age: 2–5 years old; sex: all
NCT04992832	Randomized, parallel assignment	HF with reduced ejection fraction	Multi-intravenous infusion of allogeneic UC-MSC (1 × 10^6^ cells/kg)	40 participants; age: 18–80 years old; sex: all
NCT04396899	Single group assignment, open-label	HF	Implantation of iPSC-CM and stromal cells in a bovine collagen type I hydrogel	53 participants; age: 18–80 years old; sex: all
NCT02962661	Randomized, parallel assignment	Cardiomyopathy, HF, hematopoietic and lymphoid cell neoplasm, and malignant solid neoplasm.	IV and TESI of allogeneic human BM-MSCs	72 participants; age: 18–80 years old; sex: all
NCT05068674	Randomized, sequential assignment, open-label	Chronic ischemic left ventricular dysfunction.	Multiple injections of human ESC-CMs (50, 150 or 300 × 10^6^ cells)	18 participants; age: 21–80 years old sex: all
NCT03763136	Randomized, parallel assignment, double-blind	HF	DI of allogenic human iPSC-CM, 2 × 10^8^ cells)	20 participants; age: 35–75 years old; sex: all
NCT04939077	Randomized, parallel assignment, triple-blind	Myocardial ischemia and LV dysfunction	DI of allogeneic human UC-MSC (1 × 10^7^ cells)	20 participants; age: ≤70 years old; sex: all
NCT02462330	Randomized, parallel assignment, double-blind	Chronic myocardial ischemia	DI of autologous BM-MSC (6 × 10^7^ cells)	90 participants; age: 18–75 years old; sex: all
NCT03797092	Randomized, parallel assignment, single-blind	NIDCM	Allogeneic adipose-derived stromal cells	30 participants; age: 30–80 years old; sex: all
NCT03572660	Single group assignment	Dilated cardiomyopathy	IC of autologous BMMNC and G-CSF	20 participants; age: 18–85 years old; sex: all
NCT04476901	Randomized, parallel assignment, quadruple-blind	NIDCM	TESI of allogeneic human MSC (1 × 10^8^ cells)	136 participants; age: 18–80 years old; sex: all
NCT05043610	Randomized, parallel assignment, single-blind	Acute MI, anterior wall myocardial infarction, ventricular cardiac remodeling, STEMI, and HF	IC of allogeneic UC-MSC (1 × 10^7^ cells).	240 participants; age:18–65 years old; sex: all
NCT02923609	Sequential assignment, open-label	HF with normal ejection fraction	TESI of autologous CD34+ cells	30 participants; age: 18–70 years old; sex: all
NCT01652209	Randomized, parallel assignment, open-label	Acute MI	IC of autologous BM-MSC (1 × 10^6^ cells/kg)	90 participants; age: 20–75 years old; sex: all
NCT04684602	Non-randomized, sequential assignment	Cardiovascular disorders	Allogeneic amniotic and umbilical cord stem cell	5000 participants; age: 18–75 years old; sex: all
NCT03406884	Randomized, parallel assignment, open-label	HLHS	DI of autologous c-kit+ cells—12,500 cells/kg	32 participants; age: 1–21 days old; sex: all
NCT04982081	Randomized, parallel assignment, double-blind	Cardiovascular diseases, congestive HF, and dilated cardiomyopathy	Catheter-based endocardial delivery of human iPSC-CM (1 or 4 × 10^8^ cells)	20 participants; age: 18–75 years old; sex: all
NCT04945018	Non-randomized, parallel assignment	HF and ischemic heart disease	TESI of allogeneic iPS–cell–CM spheroids suspension	10 participants; age: 18–80 years old; sex: all

AEC, amniotic/amnion epithelial cells; BMMNC, bone marrow mononuclear cells; BM-MSC, bone marrow mesenchymal stem cells; CM, cardiomyocytes; CSC, cardiac stem cells; DI, intramyocardial injection; DM, diabetes mellitus; ESC-CMs, embryonic-stem-cell-derived cardiomyocytes; G-CSF, granulocyte colony-stimulating factor; HF, heart failure; HLHS, hypoplastic left heart syndrome; IC, intracoronary infusion; iPSC, induced pluripotent stem cell; iPSC-CM, induced pluripotent stem cell derived cardiomyocytes; IC, intracoronary infusion; IV, intravenous infusion; LV, left ventricular; MI, myocardial infarction; MSC, mesenchymal stem cells; NIDCM, non-ischemic dilated cardiomyopathy; STEMI, ST-segment-elevation myocardial infarction; UC-MSC, umbilical-cord-derived mesenchymal stem cells.

## Data Availability

Not applicable.
